# Linking landscape habitats with prevalence of fusarium wilt disease of cashew crop in Tanzania

**DOI:** 10.1186/s12862-024-02284-5

**Published:** 2024-07-23

**Authors:** William V. Mbasa, Wilson A. Nene, Fortunus A. Kapinga, Stella G. Temu, Donatha D. Tibuhwa

**Affiliations:** 1 Department of Molecular Biology and Biotechnology, University of Dar es Salaam, P.O. Box 35179, Dar es Salaam, Tanzania; 2 Tanzania Agricultural Research Institute-Naliendele, P.O. Box 509, Mtwara, Tanzania

**Keywords:** Cashew, Landscape habitat, *Fusarium oxysporum*, Epidemic, Habitat-disease interactions

## Abstract

Epidemic of Cashew Fusarium wilt disease (CFWD) has been a continuous focal challenge in the cashew farming, in Tanzania. Limited to edaphic conditions as a major factor in its epidemic, the current study aimed to assess the habitat-disease relationship. Purposive surveys involving assessment of disease prevalence and habitat compositions were conducted across four landscapes of southeastern zone from 2019 to 2023. Findings revealed a widespread of CFWD across diversified landscapes possessing varying habitat characteristics, mainly cultivated land with mature cashew, brownish sand loamy soils, grassland or shrub vegetation, seasonal river streamlines and natural water wells. The highest disease incidence and severity were noted at Nachingwea/Masasi plain (99.28:88.34%) followed by Liwale inland plain (98.64:89.3%), Coastal zone (72.72:59.83%) and Tunduru dissected plain (62.13:54.54%). The habitat characteristics were strongly similar within the landscape (0.86-Jaccard index) except between villages of the coastal zone (0.71-Jaccard index). Across landscapes, Nachingwea/Masasi plains and the Coastal zone were strongly similar to Tunduru dissected plain (0.63—1.0-Jaccard index), but strongly dissimilar with the Liwale inland plain (0.67—0.70- Jaccard distance). Furthermore, the presence of greater than 0.5 suitability indices across landscapes were revealed, with Liwale inland plain having strongest suitability index of 0.743 followed by Coastal zone (0.681), Tunduru dissected plain (0.617) and Nachingwea/Masasi plain. Significantly, the habitats had an increase of 0.1 suitability index, and positively correlated with disease prevalence by triggering disease incidence of 13.9% and severity of 31.4%. The study for the first time revealed the presence of an association between disease prevalence and landscape habitat characteristics of southeastern, Tanzania; paving the way to inclusive thinking of habitat as one of the drivers in the prevalence of fusarium wilt disease of cashews. Further research on the genetic coevolution of *Fusarium oxysporum* across landscapes to strengthen disease risk management in the cashew industry is recommended.

## Introduction

*Fusarium oxysporum* is a soilborne pathogenic fungus [1]; inflicting economic losses to different host crops [2–5]. In cashew (*Anacardium occidentale* L.), the pathogen attacks inflict 100% yield and crop losses, leaving cashew field’s bare, ultimately triggering poverty among the farming community [6, 7]. The losses occur after the pathogen enter through the wounded roots, sporulate and block the xylem vessel [6–9]. Post-xylem blocking, the cashew displays leaves yellowing, browning, wilting and finally dying [6, 10]. In Tanzania, the pathogen attack was first reported in 2012 from Mkuranga district in the Coast region by smallholder farmers [10]. Lilai et al., [7], reported spread of the disease in villages of Lindi and Mtwara regions in 2019. However, further outbreaks of fusarium wilt disease of cashew have been reported from different areas within previous reported and new regions (TARIN, 2022 unpublished).

Studies have been conducted on various factors contributing to the vast spread of fusarium wilt disease in cashew in Tanzania. Our previous studies reported by Lilai et al., [7] and Tibuhwa and Shomari, [10] indicated that among many factors, edaphic factor including soil pH below 6.4, high temperature range of about 25 – 31 °C and low moisture content are the major contributing factor. Again a reports by Tibuhwa and Shomari, [10] and TARIN, (2022 unpublished) articulated that rain season water runoff, seepage and anthropogenic factors such as uses of farm equipment, clothes, footwear, tools, and containers that had been used in infested areas without disinfecting, and land cultivation causing wounds on roots have contributed to pathogen dispersal and disease epidemic. However, these previous findings are based on soil ecological conditions and anthropogenic factors per see, leaving behind other factors including landscape habitat, and biodiversity.

Landscape habitat, which comprises vegetation and geologic conditions, is an important factor contributing to the occurrence, spread and maintenance of the plant disease [11, 12]. The ecological habitat characteristics directly impact pathogen prevalence through effects on host nutrition/immune response to pathogens, meanwhile vegetation cover acts as an alternative host [11, 13]. Indirectly, ecological habitat characteristic increase/decrease the biodiversity and their competition [14, 15]. For instance, similarity of habitats has contributed to an epidemic of Fusarium wilt of banana in various areas such as Panama, Australia, and Eastern Africa [8, 16, 17]. Therefore observing the spread of Fusarium wilt disease of cashew, in different landscapes such as Makonde plateau, Coastal zones, Nachingwea Masasi Plain and Inland plains, the perspective of habitat ecology is of paramount importance. Unfortunately, little emphasis has been placed on relating landscape habitats with the epidemic of fusarium wilt disease of cashew. Therefore, this study aimed to understand the contribution of landscape habitat (habitat composition, similarities and suitability) on the prevalence of Fusarium wilt disease of cashew in Tanzania.

## Materials and methods

### Study sites

The landscape epidemiological study was conducted in landscapes of southeastern Tanzania from 2019 to 2023. The landscapes were Nachingwea/Masasi Plain, Coastal Zone, Tunduru Dissected Plain and Liwale Inland plains. The southeastern zone was selected for researches due to the presence of active infestation of Fusarium wilt disease. Southeastern zone is one of seven agricultural zones in Tanzania comprising the two regions of Mtwara (10°16′0 "S, 40°11′0" E) and Lindi (9°59′15.36′′S, 39°41′53.52′′E), and Tunduru district in Ruvuma region (11°2′26.491 "S 37°19′45.43" E). It is commonly characterized by a mean air and soil temperature of 28.0 °C and 31.0 °C respectively and a unimodal rainfall type, that starts from November/December to April/May with mean annual rainfall ranges from 800 to 1200 mm [18, 19]. The zone also has a relative humidity of 70% [7, 18, 20].

### Prevalence of fusarium wilt disease

**Fig. 1:**
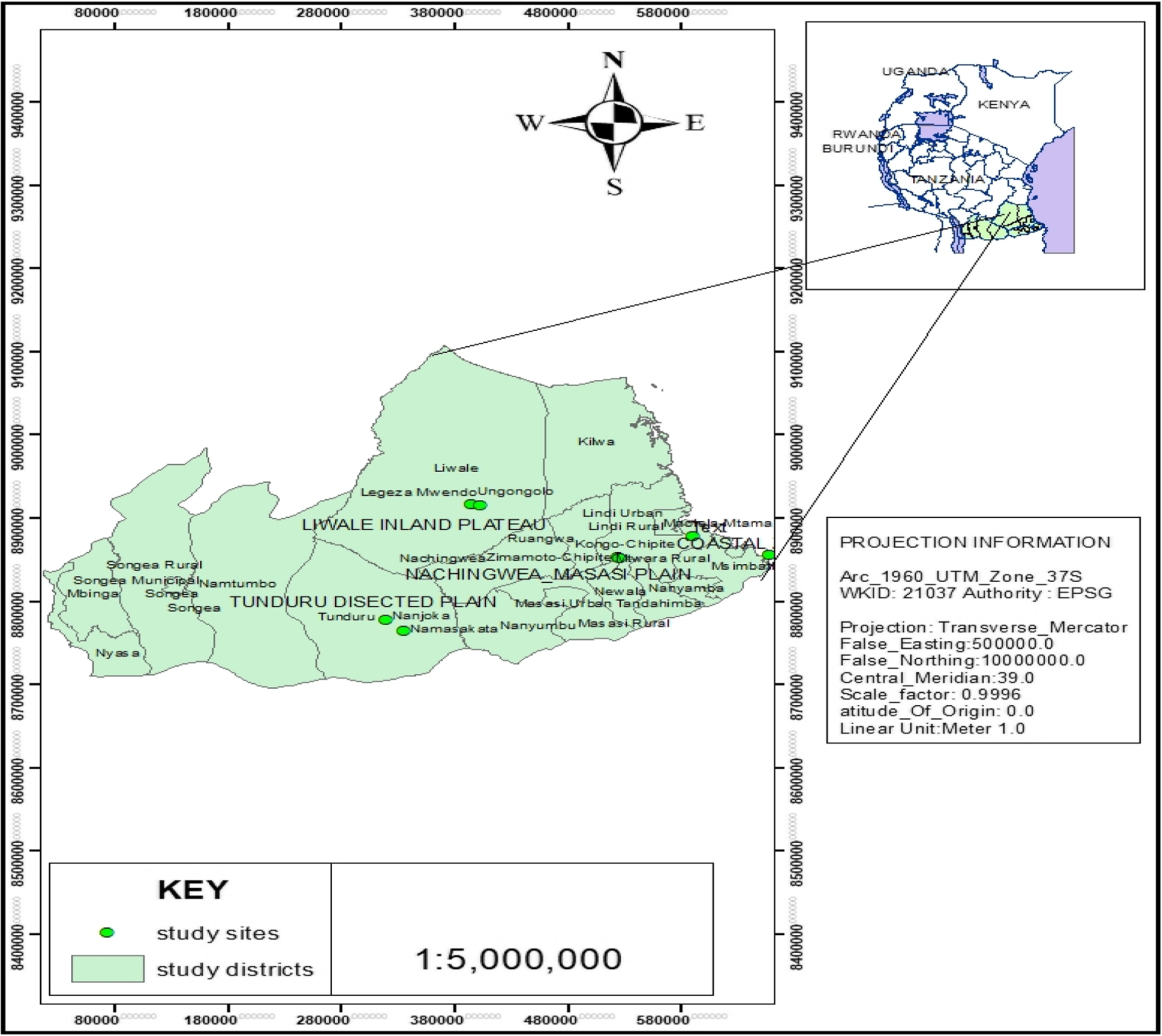
Surveyed areas infected with fusarium wilt disease in Liwale inland plain, Nachingwea/Masasi plain, Tunduru dissected plain and Coastal zone (The map is on courtesy to Mr. Zawadi Kilingala-GIS specialist)

Our first intensive field survey was conducted and reported at the end of rainy season (April to May) of 2019 within some villages of districts of Mkuranga (coastal zone), Masasi, Nachingwea (Nachingwea/Masasi Plain), Tandahimba, Newala (Makonde Plateau), and Liwale (Liwale inland plain) of the southeastern zone [7]. However, since 2020 to date, District Agricultural, Irrigation and Cooperation officers have reported infestations in new villages within existing and new landscapes (TARIN, 2022 unpublished). Consequently, we conducted an intensive survey across new reported areas occupying four landscapes (Nachingwea/Masasi Plain, Coastal Zone, Tunduru Dissected Plain and Liwale Inland Plain) during rainy season of December 2022 to March 2023. Two villages per landscapes were purposive selected and surveyed on the prevalence of Fusarium wilt disease with their geographical coordinates collected using handheld GPS (model Garmin Etrex 20x, 2.2 Inches with Distance Sensor). The surveyed villages as presented in Fig. 1, included Mnolela, Msimbati (Coastal zone), Kongo Chipite, Zimamoto-Chipite (Nachingwea/Masasi Plain), Nanjoka, Namasakata (Tunduru Dissected Plain), Legeza-Mwendo and Ungongolo (Liwale Inland Plain).

Assessment of disease prevalence was conducted by observing and recording the incidence of disease occurrence on 70 cashew trees (2.5 acres). Visual observation of the symptoms of the disease was conducted using the incidence levels; 1). Symptomatic, 2). Asymptomatic and 3). Dead cashew trees following modification of Lilai et al., [7] symptom classification. Disease severity was also assessed as a measure of disease prevalence through scoring of the leaf symptoms on symptomatic and dead cashew trees. The scoring was conducted on four cardinal points of cashew canopy i.e., north, south, east and west using the modified score scale of Mbasa et al., [6]. The observers on the incidence and severity of the diseases were subjected to Eqs. 1 and 2 respectively.

1 $$\mathrm{Disease}\,\mathrm{Incidence}\,(\%)=\frac{\mathrm{Infested}\,\mathrm{cashew}\;(\mathrm{symptomatic}\,/\mathrm{dead})}{\mathrm{Total}\;\mathrm{cashew}}\times100$$

2 $$\mathrm{Disease}\,\mathrm{Severity}\,(\%)\,=\;\frac{\sum\;(\mathrm{No}.\,\mathrm{of}\,\mathrm{scale}\,\mathrm x\,\mathrm{Percent}\,\mathrm{midpoint}\,\mathrm{range}\,\mathrm{of}\,\mathrm{scale})}{\sum\;(\mathrm{Total}\,\mathrm{number}\,\mathrm{of}\,\mathrm{counted}\,\mathrm{cashew}\,\mathrm{sides})}\times100$$

### Landscape habitat similarity and suitability

We conducted field survey during the rainy season (January to March) of 2019, 2020, 2022 and 2023 in reported areas across four landscapes observing the habitat characteristics. During surveys across years of field experience, five habitat characteristic types including; soil, vegetation, anthropogenic activities, cashew age/size and water source were used as the major landscape habitat characteristics. A line transect of 100 m was laid in perpendicular directions across cashew field. The habitat compositions of the infested cashew field were identified in consultation with experienced local experts. From five-landscape habitat characteristics, different profound variables were observed and used in determining landscape habitat similarity, dissimilarity and suitability.

Habitat similarity and dissimilarity indices were determined using the modified Jaccard index and Jaccard distance respectively following Kiernan, [21]. The Jaccard index was determined based on the number of shared characteristics (variables) between two landscapes. The variables of the landscapes were qualitatively compared, and then shared and unique variables were noted and subjected in Eq. 3 for calculating Jaccard index. After attainment of Jaccard index, which has a maximum of index of one, the Jaccard distance for habitat dissimilarity was determined using the Eq. 4.

3 $$\text{Jaccard index }(\text{SJ}) =^{c}\!\left/ \!_{(a+b+c)}\right.$$

whereby, *SJ* is the Jaccard similarity index, *c* is the number of shared variables between the two landscape habitats and *a* and *b* are the number of variables unique to each landscape habitat.

4 $$\text{Jaccard distance }= 1-SJ$$

whereby, 1.0 is the maximum value of Jaccard index, *SJ* is the Jaccard similarity index.

Landscape habitat suitability for prevalence of Fusarium wilt disease of cashew across landscapes was determined using the modified habitat suitability index of Kiernan, [21]. Habitat variables were assigned the percentage score between 0–100%, that were obtained by first determining and comparing the variables across all studied landscapes, then calculating the frequency of occurrence of individual variable in percentage (frequency of occurrence divide by total landscapes times 100). The obtained percent score of individual variable was labelled as a suitability index variant (SIv). The suitability index variant was multiplied by each other with respect to landscape to obtain the geometric mean and resulting figure was divided by the total number of suitability index variant within the landscape as per Eq. 5. Thereafter, Habitat Suitability Index (HIS) was grouped into categories from 0 to 1 as presented in Table 1.

5 $$HSI=^n\sqrt{SIv\;x\,Slv2\;x\;....\;SIvn}$$

whereby HSI is a Habitat suitability index, *Slv* is the Suitability index variant and *n* is the total number of suitability index variant.

**Table 1:** Habitat similarity and suitability index score rank

HIS categories	JS and HSI Index
Poor	< 0.5
Average	0.5 – 0.59
Strong	0.6 – 0.79
Strongest	0.8 – 1.0

### Data analysis

The collected data on disease incidence and severity across landscapes were non-parametrically analyzed using Kruskal–Wallis test. Mann–Whitney test was used for mean rank comparison of the landscape sites. Jaccard index and Jaccard distance were prepared in matrix comparison across landscapes. The relationship between habitat suitability and prevalence of cashew fusarium wilt disease (CFWD) was analyzed using Spearman correlation. Origin Pro 2019 was used for statistical analysis and Microsoft Office Excel 2021 was used for constructing matrix comparison.

## Results

### Prevalence of cashew fusarium wilt disease in four landscapes

**Fig. 2:**
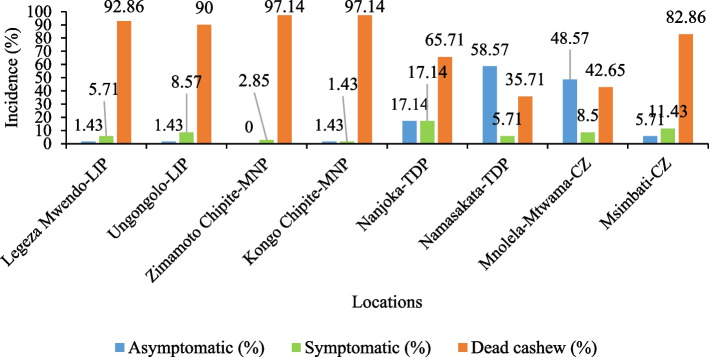
Incidence of cashew fusarium wilt disease across the landscapes of southeastern, Tanzania

**Fig. 3:**
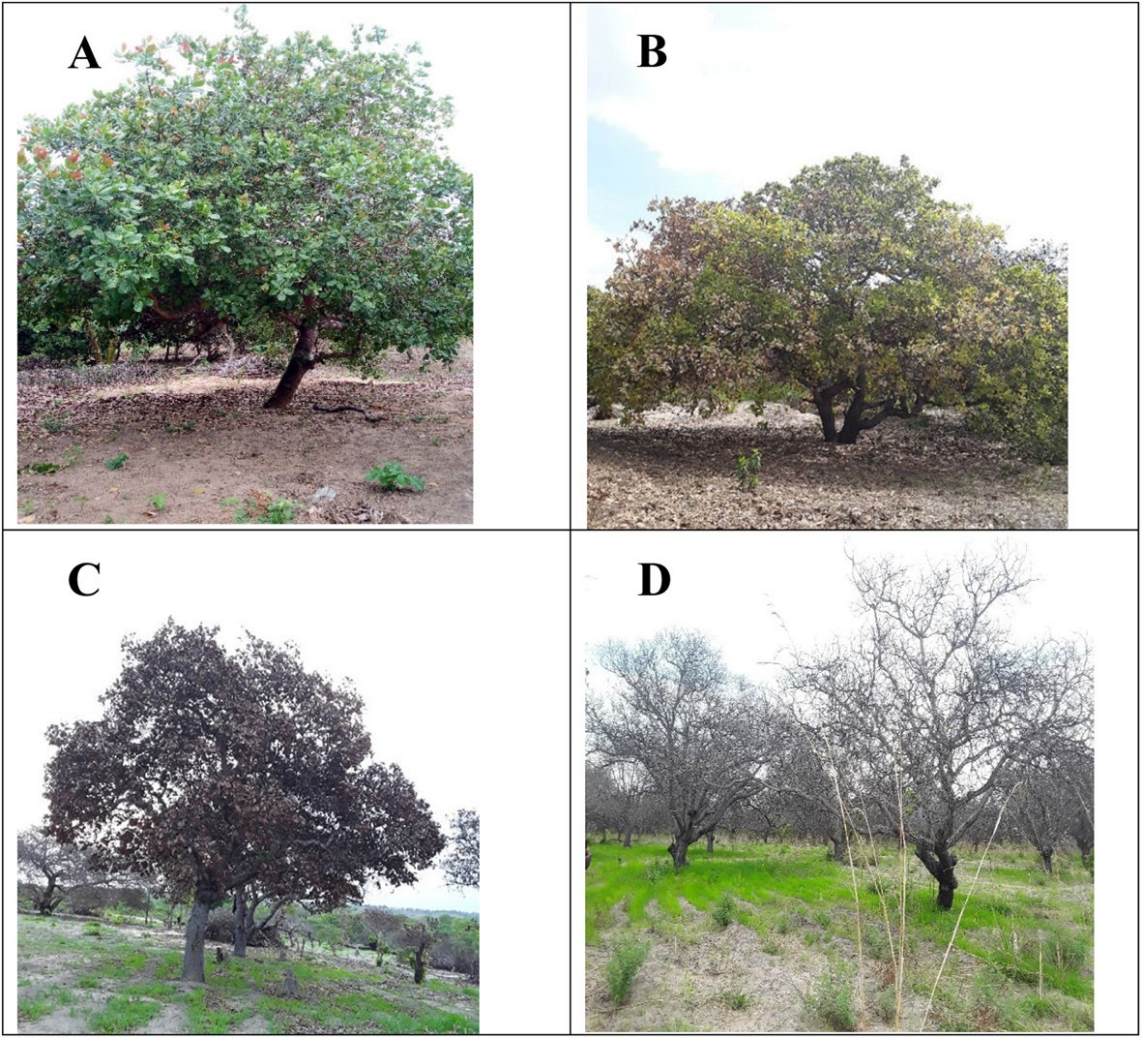
Incidence and severity of Fusarium wilt disease of cashew. **A** Asymptomatic cashew tree, **B** Symptomatic cashew tree with yellowing and brownish symptoms, **C** Wilted Cashew tree, and **D** Abandoned cashew field with cashew logs after infestation of Fusarium wilt disease (The photos are on courtesy to Mr. William V. Mbasa)

Cashew Fusarium wilt disease prevailed across landscapes of the Southeastern zone as presented in Figs. 2 and 3. Significant variation was noted among the villages attacked with CFWD (*Chi-square probability* < *. 001*). Villages in Nachingwea/Masasi plain had high average disease incidence of 99.28% similar to Liwale inland plain (98.64%) compared to Coastal zones (72.72%) and Tunduru dissected plain (62.13%). A high number of wilted and dead cashew trees (Fig. 3C & D) were noted at Zimamoto and Kongo Chipite (97.14%) trailed by Legeza Mwendo (92.86%), Ungongolo (90%), and Msimbati (82.86%). For symptomatic cashew trees (Fig. 3B), highest incidence was recorded at Nanjoka (17.4%), followed by Msimbati (11.43%), Mnolela (8.5%) and Ungongolo (8.5%). While for an asymptomatic cashew tree (Fig. 3A), among other, Namasakata was less affected with 58.57% asymptomatic trees followed by Mnolela (48.57%) in coastal zone and Nanjoka (17.14%) in Tunduru dissected plain.

Assessment of disease severity displayed significant variation among villages across landscapes as presented in Table 2 (Chi square probability < 0.001, H-value = 96.17, Sample size = 69). The villages of Liwale inland plain had averaged higher disease severity of 89.3% nexted with Nachingwea/Masasi plain (88.34%), Coastal zone (59.83%) and least of Tunduru dissected plain with 54.54. Village wise, higher disease severity was noted from Ungongolo (90.61%), Legeza Mwendo (88.06%), Zimamoto Chipite (88.54%), Kongo Chipite (88.14%) and Msimbati (84.02%) compared to others. Mnolela village from Coastal zone possessed low disease severity of 35.64% tailed by Namasakata (46.47%) and other villages. Pairwise comparison revealed that Legeza Mwendo, Ungongolo, Zimamoto Chipite, Kongo Chipite and Msimbati villages were significantly different from Nanjoka, Namasakata and Mnolela (*P-value* < 0.001), but insignificant among themselves (*P-value* > 0.05, Table 3). Yet again, no significant pairwise comparison was noted among villages within the same landscape (*P*-value > 0.05) except for Msimbati and Mnolela from the Coastal zone (*P-value* < 0.001, U-Value = 1058.5).

**Table 2:** Severity of cashew fusarium wilt disease (Kruskal–Wallis test) across landscapes of Southeastern, Tanzania

Landscapes	Locations	Size	Disease severity (%)	H Value	Chi-square probability
Liwale Inland Plain	Legeza Mwendo	69	88.06	96.17	< 0.001
	Ungongolo	69	90.61		
Masasi/Nachingwea Plain	Zimamoto-Chipite	69	88.54		
	Kongo-Chipite	69	88.14		
Tunduru Dissected Plain	Nanjoka	69	62.62		
	Namasakata	69	46.47		
Coastal Zone	Mnolela	69	35.64		
	Msimbati	69	84.02

**Table 3:** Mann–whitney comparison on severity of cashew fusarium wilt disease across four landscapes of Southeastern, Tanzania

Location Comparison	Sample size	Mann–Whitney U-Value	*P-value*	Disease severity (%)
Legeza Mwendo LIP vs. Ungongolo LIP	69 vs. 69	2346.5	0.885	88.06 vs. 90.61
Legeza Mwendo LIP vs Kongo Chipite MNP	69 vs. 69	2347.5	0.888	88.06 vs. 88.54
Legeza Mwendo LIP vs. Nanjoka TDP	69 vs. 69	1607	< 0.001	88.06 vs. 88.14
Legeza Mwendo LIP vs. Namasakata TDP	69 vs. 69	1212.5	< 0.001	88.06 vs. 62.62
Legeza Mwendo LIP vs. Mnolela CZ	69 vs. 69	928	< 0.001	88.06 vs. 46.47
Legeza Mwendo LIP vs. Msimbati CZ	69 vs. 69	2209	0.465	88.06 vs. 35.64
Legeza Mwendo LIP vs Zimamoto Chipite MNP	69 vs. 69	2346.5	0.885	88.06 vs. 84.02
Ungongolo LIP vs. Zimamoto Chipite MNP	69 vs. 69	2378.5	0.993	90.61 vs. 88.54
Ungongolo LIP vs. Kongo Chipite MNP	69 vs. 69	2378.5	0.993	90.61 vs. 88.14
Ungongolo LIP vs. Nanjoka TDP	69 vs. 69	1567.5	< 0.001	90.61 vs. 62.62
Ungongolo LIP vs. Namasakata TDP	69 vs. 69	1174.5	< 0.001	90.61 vs. 46.47
Ungongolo LIP vs. Mnolela CZ	69 vs. 69	887.5	< 0.001	90.61 vs. 35.64
Ungongolo LIP vs. Msimbati CZ	69 vs. 69	2172	0.375	90.61 vs. 84.02
Zimamoto Chipite MNP vs Kongo Chipite MNP	69 vs. 69	2379.5	0.997	88.54 vs. 88.14
Zimamoto Chipite MNP vs Nanjoka TDP	69 vs. 69	1573.5	< 0.001	88.54 vs. 62.62
Zimamoto Chipite MNP vs Namasakata TDP	69 vs. 69	1178.5	< 0.001	88.54 vs. 46.47
Zimamoto Chipite MNP vs Mnolela CZ	69 vs. 69	895.5	< 0.001	88.54 vs. 35.64
Kongo Chipite MNP vs Nanjoka TDP	69 vs. 69	1584	< 0.001	88.14 vs. 62.62
Kongo Chipite MNP vs Namasakata TDP	69 vs. 69	1196	< 0.001	88.14 vs. 46.47
Kongo Chipite MNP vs Mnolela CZ	69 vs. 69	915.5	< 0.001	88.14 vs. 35.64
Kongo Chipite MNP vs Msimbati CZ	69 vs. 69	2175.5	0.383	88.14 vs. 84.02
Nanjoka TDP vs Namasakata TDP	69 vs. 69	1936	0.058	62.62 vs. 46.47
Nanjoka TDP vs Mnolela CZ	69 vs. 69	1622	0.001	62.62 vs. 35.64
Nanjoka TDP vs Msimbati CZ	69 vs. 69	1766	0.009	62.62 vs. 84.02
Namasakata TDP vs Mnolela CZ	69 vs. 69	2067	0.182	46.47 vs. 35.64
Namasakata TDP vs Msimbati CZ	69 vs. 69	1356.5	< 0.001	46.47 vs. 84.02
Mnolela CZ vs Msimbati CZ	69 vs. 69	1058.5	< 0.001	35.64 vs. 84.02

### Landscape habitat composition and similarities

**Fig. 4:**
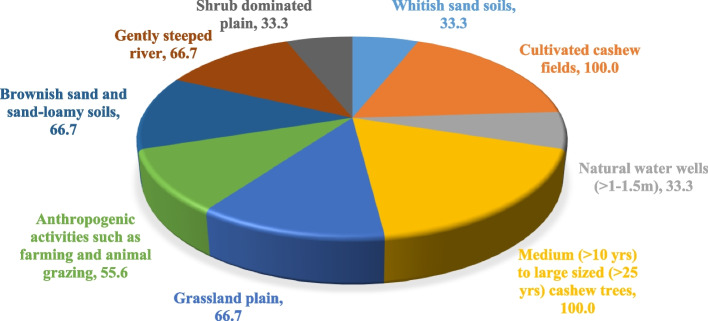
Habitat composition (%) across landscapes of southeastern Tanzania

**Fig. 5:**
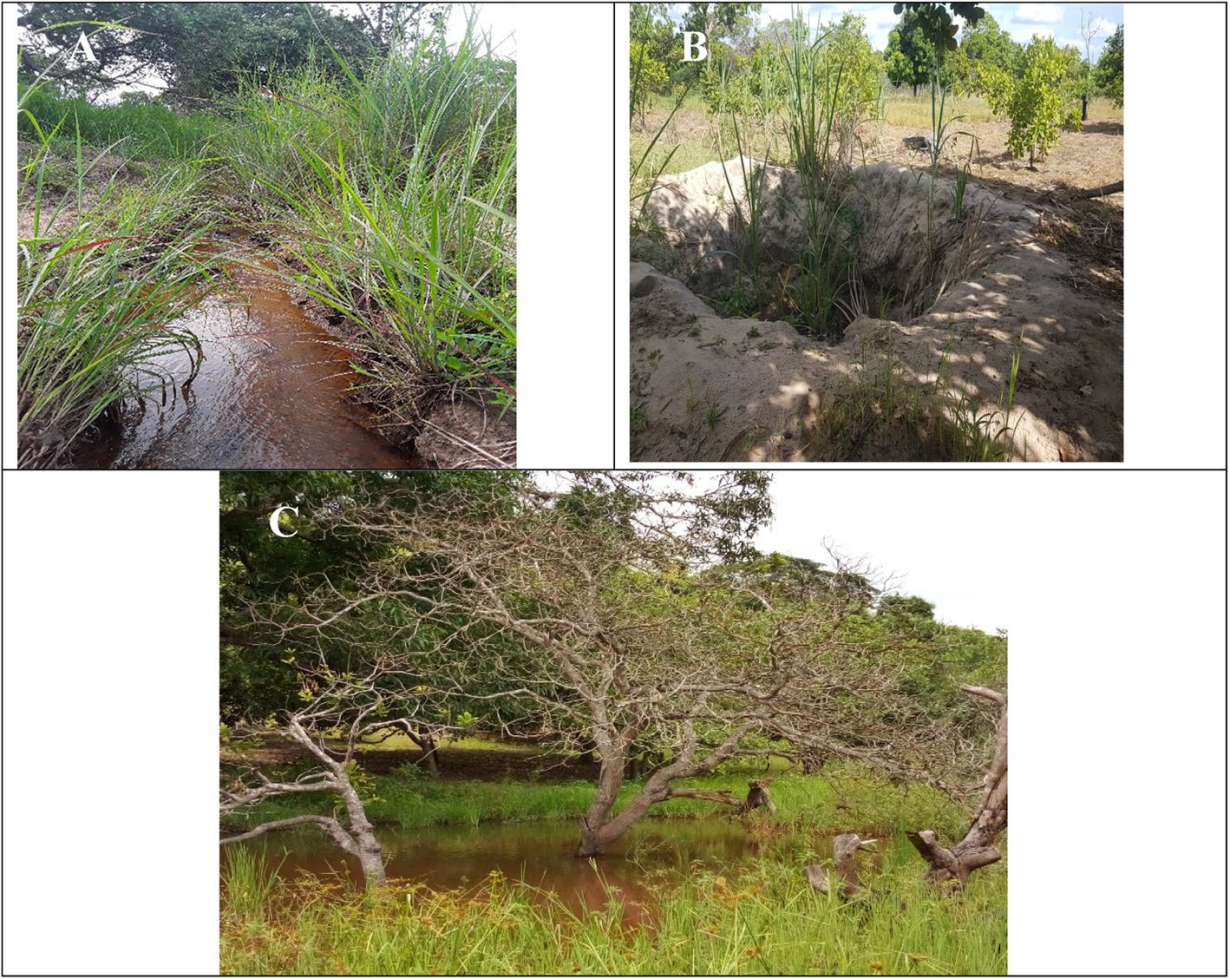
Landscape habitat features of infested cashew fields **a** Seasonal river stream at Nanjoka-Tunduru dissected plain, **B** Natural water well at Liwale inland plain and **C** Seasonal river stream at Chipite-Masasi/Nachingwea plain. (The photos are on courtesy to Mr. William V. Mbasa)

The results of landscape habitats displayed varied composition of characteristics across the landscapes of southeastern zones as presented in Fig. 4 and Table 4. Cashew fields across landscape villages were 100% dominated with matured cashew (in between ten (10) years and twenty-five (25) years of age). Anthropogenic activities including farming of various crops such as sesame, pigeon pea, cowpea, vegetables, and livestock grazing occupied 55.6% of variable characteristics presenting in landscape of Ungongolo-Liwale inland plain, Namasakata in Tunduru dissected plain and Kongo-Chipite in Nachingwea/Masasi plain. Grasslands occupied 66.7% of habitat characteristic in the village of Liwale inland plain, Tunduru dissected plain and the Coastal zone, tailed by shrub-domination (33.3%) in Nachingwea/Masasi plain. Brownish sand/sand loamy soils with 66.7% habitat characteristics was occupied in the villages of Tunduru dissected plain, Nachingwea/Masasi plain and Mnolela in Coastal zone, compared to whitish sand soils (33.3%) in Liwale inland plain and Msimbati in Coastal zone. On the other hand, water source composition displayed the presence of seasonal river streamlines with gentle steepness (66.7%) and natural water wells (> 1–1.5 m) occupying 33.3% (Fig. 5).

**Table 4:** Habitat characteristic composition of four landscapes in southeastern, Tanzania

Habitat characteristics	Location							
	Ungongolo-Liwale	Legeza Mwendo-Liwale	Namasakata-Tunduru	Nanjoka-Tunduru	Kongo-Chipite-Masasi	Zimamoto-Chipite-Masasi	Mnolela-Mtama-Lindi	Msimbati-Mtwara Rural
Whitish sand soils	** + **	** + **	**-**	**-**	**-**	**-**	**-**	** + **
Cultivated cashew fields	** + **	** + **	** + **	** + **	** + **	** + **	** + **	** + **
Natural water wells (> 1–1.5 m)	** + **	** + **	**-**	**-**	**-**	**-**	**-**	** + **
Medium (> 10 yrs.) to large sized (> 25 yrs.) cashew trees	** + **	** + **	** + **	** + **	** + **	** + **	** + **	** + **
Grassland plain	** + **	** + **	** + **	** + **	**-**	**-**	** + **	** + **
Anthropogenic activities such as farming and animal grazing	** + **	**-**	** + **	**-**	** + **	**-**	**-**	**-**
Brownish sand and sand-loamy soils	**-**	**-**	** + **	** + **	** + **	** + **	** + **	**-**
Gently steeped river	**-**	**-**	** + **	** + **	** + **	** + **	** + **	**-**
Shrub dominated plain	**-**	**-**	**-**	**-**	** + **	** + **	**-**	**-**
Coordinate Geometry (GPS)	*09˚47′40.4’’ S 038˚02′02.9’’E*	09˚48′45.1’’ S 038˚06′32.9’’E	11˚10′0’’ S 37˚29′0’E	11°03′28.3"S 37°20′25.1"E	10°22′20.7"S 39°13′1.1"E	10°23′06.7"S 39°13′36.2"E	10°09′00"S 39°49′00"E	10°21′0"S 40°26′0"E

Key: Present ( +), Absent (-)

Surveyed landscapes displayed similarities of habitat characteristics (Table 5). Infested areas within the same landscape showed strong similarities (Jaccard index ranging from 0.71 to 0.86) compared with across landscapes (Jaccard index ranging from 0.3 to 0.75). Ungongolo and Legeza Mwendo occupying Liwale inland plain, Nanjoka, Namasakata (Tunduru dissected plain), Kongo-Chipite, and Zimamoto-Chipite (Masasi/Nachingwea Plain) had strongest Jaccard similarity index of 0.86 compared to the 0.71-Jaccard index of Mnolela and Msimbati villages from Coastal zone. Across landscapes, Mnolela (Coastal zone) was strongly similar with Nanjoka (Jaccard index = 1.0) and Namasakata (Jaccard index = 0.86) from Tunduru dissected plain. Again, Kongo-Chipite and Zimamoto-Chipite from Masasi/Nachingwea plain possessed strong similar habitat characteristics with Nanjoka and Namasakata from Tunduru dissected plain (0.75, 0.63 and 0.63, 0.71). On the other hand, dissimilarity ranging from 0.14 to 0.70 was noted on infested areas across the landscapes (Table 6). Ungongolo and Legeza Mwendo (Liwale inland plain) had strong dissimilarity index of 0.70 with Zimamoto and Kongo-Chipite in Masasi/Nachingwea plain. Furthermore, strong Jaccard dissimilarity was observed between Zimamoto-Chipite (Masasi/Nachingwea plain) with Msimbati in coastal zone and Legeza Mwendo in Liwale inland plain (Jaccard distance = 0.67).

**Table 5:** Habitat similarities matrix (Jaccard Similarity Index) of infested cashew fields in four landscapes

Location	UL	LML	NST	NJT	KCM	ZCM	MML	MMR
UL	1.00							
LML	0.86	1.00						
NST	0.56	0.44	1.00					
NJT	0.44	0.50	0.86	1.00				
KCM	0.40	0.30	0.75	0.63	1.00			
ZCM	0.30	0.33	0.63	0.71	0.86	1.00		
MML	0.44	0.44	0.86	1.00	0.63	0.71	1.00	
MMR	0.86	1.00	0.44	0.50	0.30	0.33	0.50	1.00

Key: *UL* Ungongolo-Liwale, *LML* Legeza Mwendo-Liwale, *NST* Namasakata-Tunduru, *NJT* Nanjoka-Tunduru, *KCM* Kongo Chipite-Masasi, *ZCM* Zimamoto Chipite-Masasi, *MML* Mnolela-Lindi, *MMR* Msimbati-Mtwara

**Table 6:** Habitat dissimilarities matrix (Jaccard Distance) of infested location across four landscapes

Location	UL	LML	NST	NJT	KCM	ZCM	MML	MMR
UL	0.00							
LML	0.14	0.00						
NST	0.44	0.56	0.00					
NJT	0.56	0.50	0.14	0.00				
KCM	0.60	0.70	0.25	0.38	0.00			
ZCM	0.70	0.67	0.38	0.29	0.14	0.00		
MML	0.56	0.56	0.14	0.00	0.38	0.29	0.00	
MMR	0.14	0.00	0.56	0.50	0.70	0.67	0.50	0.00

Key: *UL* Ungongolo-Liwale, *LML* Legeza Mwendo-Liwale, *NST* Namasakata-Tunduru, *NJT* Nanjoka-Tunduru, *KCM* Kongo Chipite-Masasi, *ZCM* Zimamoto Chipite-Masasi, *MML* Mnolela-Lindi, *MMR* Msimbati-Mtwara

### Landscape habitat suitability for CFWD prevalence

Landscape habitats displayed suitability on prevalence of Fusarium wilt disease as presented in Table 7. Noted habitat suitability indices varied among the landscapes, with Liwale inland plain strongly suitable compared to other landscapes. Respectively, villages of Liwale inland plain; Ungongolo and Legeza Mwendo had 0.743, and 0.681 highest indices followed by Namasakata in Tunduru dissected plain (0.617), Msimbati in Coastal zone having 0.681 index and others (0.556, 0.580, 0.519, 0.556). The noted suitability indices corresponded with presence of the high incidence (41 to 99%) and severity (35.64 to 90.61%) of fusarium wilt disease of cashew across landscapes. Moreover, the suitability of landscapes was noted from 24 to 691-masl altitude.

**Table 7:** Landscape habitat suitability for fusarium wilt disease prevalence

Landscape Ecologies	SHI (0–1)	Disease Incidence (%)	Disease Severity (%)	Altitude (masl)	Class
Ungongolo-Liwale (LIP)	0.743	98.57	90.61	452	Strong suitability
Legeza-Mwendo-Liwale (LIP)	0.681	98.37	88.06	401	Strong suitability
Namasakata-Tunduru (NTDP)	0.617	41.42	46.47	568	Strong Suitability
Nanjoka-Tunduru (TDP)	0.556	82.85	62.62	691	Average Suitability
Kongo-Chipite-Masasi (NMP)	0.580	98.57	88.14	210	Average Suitability
Zimamoto-Chipite-Masasi (NMP)	0.519	99.00	88.54	228	Average Suitability
Mnolela-Mtama-Lindi (CZ)	0.556	51.15	35.64	175	Average Suitability
Msimbati-Mtwara Rural (CZ)	0.681	94.29	84.02	24	Strong Suitability

*masl* meter above sea level

**Fig. 6:**
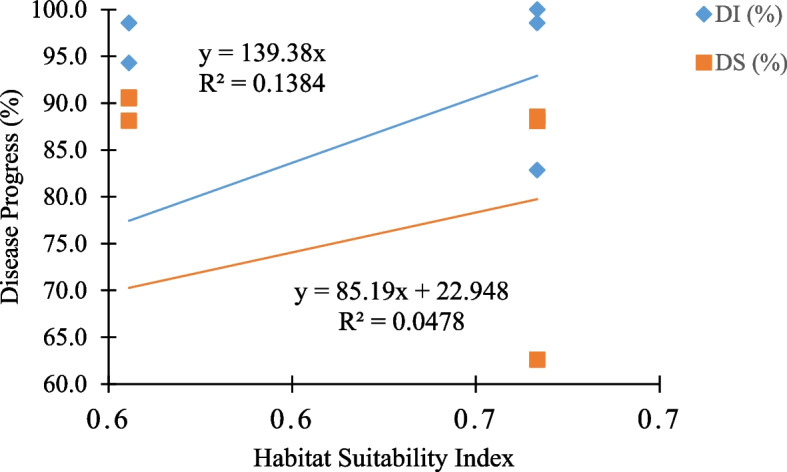
Relationship of habitat suitability index, disease incidence (%) and severity (%) across four landscapes

The relationship between disease prevalence and habitat suitability of landscapes showed significant positive Spearman correlation (Fig. 6). The suitability index was weak positively correlated with disease incidence (R2 = 0.1384, *p* < 0.001) and disease severity (R2 = 0.0478, *p* < 0.001). A change in disease incidence across landscapes was a 139.38 factor of habitat suitability index. Yet again, a change of disease severity within a landscape was a 85.19 factor of habitat suitability index with a gradient increase of 22.948.

## Discussion

Epidemics of Fusarium wilt disease of cashew were studied across landscapes of southeastern Tanzania, which included Liwale inland plain, Nachingwea/Masasi plain, Tunduru, dissected plain and Coastal zone. The prevalence of fusarium wilt disease was displayed in all studied landscapes and respective villages. Similarly, Lilai et al., [7] and Tibuhwa & Shomari, [10] reported the occurrence of fusarium wilt disease in Nachingwea/Masasi plain, Liwale inland plain, Makonde plateau and Coastal zone except Tunduru dissected plain. The spread of disease to Tunduru dissected plain, which was not previously reported; provide intuition of further outbreak in other cashew growing areas not only in Tanzania but also in other part of the world [22]. Nevertheless, the prevalence varied across the villages in landscapes; high disease incidence and severity were exhibited in villages of Nachingwea/Masasi plain (99:88%) and Liwale inland plain (98:89%), trailed by Coastal zone (72.5:60%) and Tunduru dissected plain (61:54%). Moreover, high incidence of dead cashew trees was noted as compared to symptomatic and asymptomatic in villages of Liwale inland plain, Nachingwea/Masasi plain, followed by Msimbati in Coastal zone and lasted with Tunduru dissected plain. Suggesting that, the attacking pathogen is highly virulent, leading to the death of cashew, farms remain with log of cashew, bare and ultimately abandoned by farmers [10, 23]. Variation in the incidence and severity of Fusarium wilt disease across different areas has been congruently conveyed in different host crops. For instance, studies on the epidemic of fusarium wilt of oil palm crop [24, 25], cotton [26] and banana [16] revealed wide distribution in various geographic conditions, presence of logs remnants and abandoned fields.

Ecological edaphic factors such as soil/air temperature, moisture contents, pH and others have gained much research attention as main factors attributing to the prevalence of Fusarium wilt disease in various crops including cashew [7, 22, 27]. Human activities comprising animal grazing, cultivation, movement of vehicles and uses/reuses of equipment’s are among the major factors influencing widespread dissemination of *Fusarium oxysporum* of cashew and other host crops [10, 17, 28]. Landscape habitats have been given little research attention as the major factor contributing to the prevalence of various diseases including fusarium wilt disease [29, 30]. Providentially, this study for the first time revealed the presence of an association between disease prevalence and landscape habitat characteristics of southeastern, Tanzania. The landscapes possessed varying habitat characteristics including; Land cultivation having mature cashew (100% characteristics composition), and intercropping of sesame, pigeon pea, vegetables and other (55.6% characteristics composition). Fusarium species including *Fusarium oxysporum* found abundant and diverse in cultivated soils with high prevalence rather than in dormant soils [31, 32]. Varying types of soil including brownish sand/sand loamy and whitish sandy soils, which respectively occupied 66.7 and 33.3%, were also a composition of landscape habitat characteristics. Vegetation cover of grassland and shrubs dominated the landscape habitats with 66.7 and 33.3% respectively. Sand or sand loamy together with grassland and shrub vegetation possess poor fertility, low microbial composition and low water retention ability, triggering alteration of host immunity and widespread of fusarium wilt disease [8, 14, 33]. Furthermore, presence of water sources including seasonal river streamlines (66.7%) and natural water wells at 1 – 1.5 m depth (33.3%) possible influences the prevalence of fusarium wilt disease. Adequate moisture contents and heated water which injures plant roots, influence fungal sporulation and penetration to host crop respectively, ultimately occurrence of disease [34–36].

Studied landscapes exhibited similarities in possession of habitat characteristics. Within landscapes, villages of Liwale inland plain, Tunduru dissected plain and Nachingwea/Masasi Plain had the strongest similar habitat characteristics (Jaccard index of 0.86) followed by 0.71-Jaccard index of villages from Coastal zone. Across landscapes, Mnolela (Coastal zone) was strongly similar to Nanjoka and Namasakata from Tunduru dissected plain (Jaccard index = 1.0, 0.86), villages from Nachingwea/Masasi plain possessed strongly similar habitat to those of Tunduru dissected plain. The similarity between these landscapes ascribes to their proximity compared to Liwale inland plain and far-coastal zone [19, 20]. Suggesting that, recorded incidence and severity of fusarium wilt disease across these landscapes is an attribution of their habitat characteristic similarities [37, 38]. On the other hand, noted strong dissimilarity between villages across landscapes of the Liwale inland plain and Nachingwea/Masasi plain (0.70 indexes), implies that the *Fusarium oxysporum* possesses ecological diverse nature with a competitive advantage in surviving [12, 30]. Congruently, *Fusarium oxysporum* is described as ecological diverse pathogen attacking both tropical and subtropical areas [39, 40]. Ecological diversity of *Fusarium* pathogen is also ascribed through its genetic variability (from formae specials to races) and host shifting ability as noted by Adeniyi et al., [41] and Alexander, [42]. Henceforth, the findings raise research needs on determining the possible occurrence of either single or multiple strains of *Fusarium oxysporum* across studied landscapes.

Different studies have displayed that suitability of ecological habitats across different landscapes influence the prevalence of plant diseases including Fusarium wilt [15, 43]. Current findings revealed the presence of greater than 0.5 suitability indices across landscapes. Based on the composition of habitats across landscapes, Ungongolo and Legeza Mwendo in Liwale inland plain respectively had 0.743 and 0.681 highest indices tailed by Namasakata in Tunduru dissected plain (0.617), Msimbati in Coastal zone (0.681) and others. Inferring that the composition of respective landscape habitats regardless of their homogeneity or heterogeneity, is one driver of prevalence of Fusarium wilt disease [27, 44]. For instance; presence of cultivated mono-host density of cashew across landscapes is one suitable habitat characteristics that contribute to the development of severe epidemics of fusarium wilt disease through roots interlocking of plant to plant [12, 45–48]. Sandy soil types contain less organic matter contents, less mineralization and microorganisms, favors less antagonism and ultimately flourishing of *Fusarium oxysporum* [44, 49, 50]. Water sources that influence the presence of moisture contents and their heating cause wounds to the host crop roots again provide a suitable habitat for disease outbreaks [25, 34, 36]. In fact, studies have indicated that landscape habitat characteristics trigger host shifting of novel pathogens and genetic coevolution leading to disease outbreaks [14, 51]. This scenario describes the reason of outbreak of fusarium wilt disease in 2012 and further prevalence to new reported areas across landscapes. Therefore, a piece of knowledge on suitability of disease-habitat relationship enhances in mapping the geographical locations for possible outbreak and prevalence of cashew fusarium wilt disease.

Furthermore, indicated significant positive correlation between disease prevalence and habitat suitability signifies the contribution of landscape habitat to the spread of fusarium wilts. An increase in disease incidence and severity is attributed to an increase in the suitability of landscape habitats and vice versa. Suggesting that, the more the suitable landscape habitat composition, the greater chance of disease to prevail, congruently to the findings of Thi Nguyen et al., [52] and Willis et al., [53]. In addition, the noted low R-squared values imply that HIS is crucial contributor of prevalence of fusarium wilt disease together with other unstudied factors such as inoculum load. On the other hand, a mathematical interpretation of disease-habitat relationship revealed a change in incidence and severity of fusarium wilt disease as a function of 139.38 and 85.19 + 22.9 factors of habitat suitability respectively. Inferring that, presence of 0.1 indices of suitable habitat across landscape, attribute to the respective occurrence of 13.9 and 31.4% of the incidence and severity of fusarium wilt disease. Using a determined mathematical relationship between disease and habitat suitability is an important component of risk rating systems [54, 55].

## Conclusion and recommendation

Current findings point out that landscape habitat is an important driver in the epidemic of Fusarium wilt disease of cashew. The presence of a continuous widespread epidemic of fusarium wilt and strong relationships of habitat-disease was noted across the landscapes of Southeastern, Tanzania. Liwale inland plain, Nachingwea/Masasi plain and the Coastal zone possessed high disease incidence and severity followed by Tunduru dissected plain. The landscapes comprised varying habitat characteristics, mainly with cultivated land having mature cashew, brownish sand/sand loamy soils, grassland/shrub vegetation, seasonal river streamlines and natural water wells (1 – 1.5 m depth). The habitat characteristics within the landscape displayed strong similarities, also across landscapes, villages of Nachingwea/Masasi plains and Coastal zone were strongly similar with Tunduru dissected plain. Again, all studied landscapes displayed strong suitability with a positive correlation between disease prevalence and suitability index across southeastern zone of Tanzania; although Liwale inland plain and Tunduru dissected plain had the strongest suitability indices. Therefore, this research proposes the utilization of landscape habitat characteristics, including seasonal river streamlines, natural water wells, brownish sand/loamy and whitish sand soils, and grassland/shrub vegetation, as predictors of disease outbreak and as criteria for selecting suitable locations for cashew farms. These findings also highlight the significance of implementing preventive measures for fusarium wilt disease. Additionally, it is crucial to conduct further investigations into the genetic coevolution of *Fusarium oxysporum* across landscapes, as this knowledge will greatly enhance disease risk management in the cashew industry.

## Data Availability

All data generated or analysed during this study is included as electronic materials and will be available at https://figshare.com.
